# Supplementary figures and images for: Anti-Dengue Activity of Lipophilic Fraction of Ocimum basilicum L. Stem
Source: Molecules. 2023 Feb 2;28(3):1446. doi: 10.3390/molecules28031446 (PMC9921342; doi:10.3390/molecules28031446)

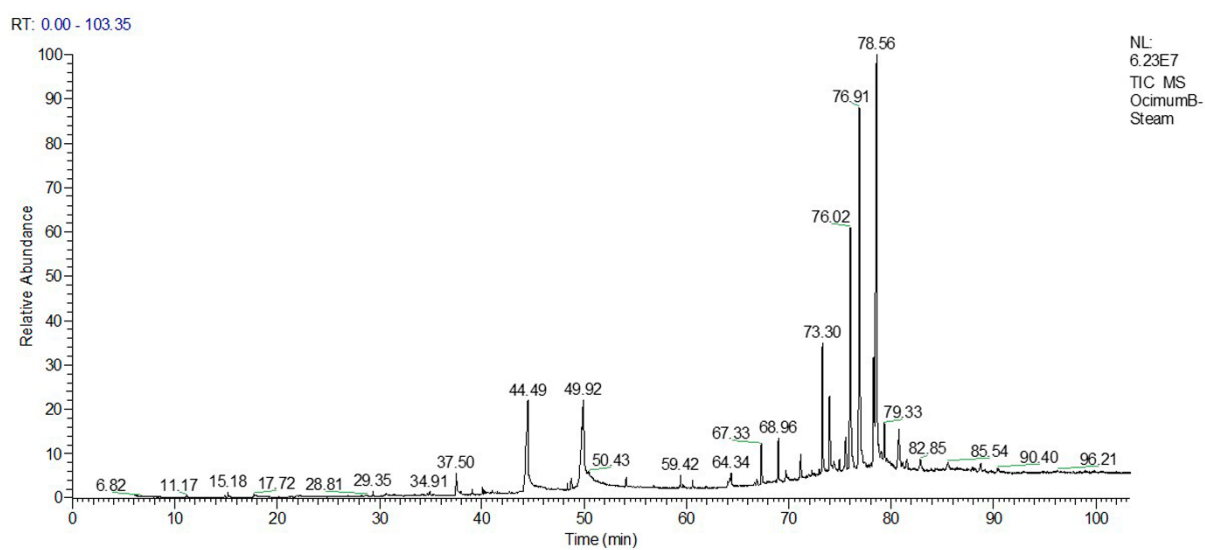

**Figure S1.** GC-TIC chromatogram of lipophilic fraction of *O. basilicum* stem.

Supplement: Supplementary file 1 [file molecules-28-01446-s001.zip › molecules-2106367-supplementary.pdf]
